# Bacterial Metabolic Activity of High-Mountain Lakes in a Context of Increasing Regional Temperature

**DOI:** 10.3390/microorganisms13061375

**Published:** 2025-06-13

**Authors:** Boyanka Angelova, Ivan Traykov, Silvena Boteva, Martin Tsvetkov, Anelia Kenarova

**Affiliations:** 1 Department of Ecology and Environmental Protection, Faculty of Biology, Sofia University “St. Kliment Ohridski”, 8 Dragan Tzankov Blvd., 1164 Sofia, Bulgaria; b_angelova@biofac.uni-sofia.bg (B.A.); itraykov@biofac.uni-sofia.bg (I.T.); kenarova@biofac.uni-sofia.bg (A.K.); 2 Department of Inorganic Chemistry, Faculty of Chemistry and Pharmacy, Sofia University “St. Kliment Ohridski”, 1 J. Bourchier, 1164 Sofia, Bulgaria; nhmt@chem.uni-sofia.bg

**Keywords:** global warming, glacial lakes, bacterial metabolic shifts, Biolog EcoPlate^TM^, bacterial functional diversity

## Abstract

Global warming poses a significant threat to lake ecosystems, with high-mountain lakes being among the earliest and most severely impacted. However, the processes affecting water ecology under climate change remain poorly understood. This study investigates, for the first time, the effects of regional warming on three high-mountain lakes, Sulzata, Okoto and Bubreka, located in the Rila Mountains, Bulgaria, by examining shifts in bacterial metabolic capacity in relation to the rate and range of utilizable carbon sources using the Biolog EcoPlate™ assay. Over the last decade, ice-free water temperatures in the lakes have risen by an average of 2.6 °C, leading to increased nutrient concentrations and enhanced primary productivity, particularly in the shallowest lake. Bacterial communities responded to these changes by increasing their metabolic rates and shifting substrate preferences from carbohydrates to carboxylic acids. While the utilization rates of some carbon sources remained stable, others showed significant changes—some increased (e.g., D-galactonic acid γ-lactone and itaconic acid), while others decreased (e.g., α-D-lactose and D-xylose). The most pronounced effects of warming were observed in June, coinciding with the onset of the growing season. These findings suggest that rising temperatures may substantially alter bacterial metabolic potential, contributing to a long-term positive feedback loop between lake nutrient cycling and climate change.

## 1. Introduction

High-mountain lakes are extreme ecosystems that are subject to harsh climatic conditions. Often regarded as sentinels of environmental change, these lakes provide valuable insights into the impacts of climate change, remote pollution, and human activities [1,2].

A significant threat to high-mountain lakes, particularly those situated above the tree line, is global warming, which accelerates snowmelt, disrupts natural hydrological patterns, hastens glacier depletion—essential for water supply to these lakes [3,4] and alters water chemistry [5]. Changes in the physical environment profoundly affect various functions within aquatic ecosystems, including metabolic rates, primary productivity dynamics, and the entire food web [6]. In many cases, these alterations can lead to losses in both structural and functional biodiversity [5,7].

High-mountain lakes typically harbor simple biological communities, with bacterioplankton playing a key role in ecosystem functioning [8,9]. Since the importance of bacteria in ecosystem functioning was recognized [10], limnologists have increasingly focused on investigating the microbial loop of the lakes’ food webs. Changes in bacterial communities can trigger cascading effects on higher trophic levels and can significantly impact the overall biogeochemical processes of the lakes. Numerous studies have documented shifts in bacterial composition in response to environmental changes [11,12,13]. However, there is a notable lack of research examining how environmental alterations affect bacterial functioning. Analyzing microbial community composition alone may not adequately reflect the impact of changes on ecosystem functionality [14,15]. For instance, the authors of [16] assessed changes in bacterial community functions using genetic profiling and Biolog EcoPlate^TM^ tests, concluding that no direct correlation exists between community structure and functioning. Several authors [14,17,18] suggest that this lack of correlation is a result of the phenotypic adaptability of microorganisms to environmental changes without corresponding alterations in community structures. This high ecological tolerance of microorganisms is attributed to their genetically determined plasticity and environmentally induced redundancy.

One of the easiest approaches for testing microbial tolerance to environmental changes is to elucidate their functional responses through shifts in their metabolic activities. Numerous studies have utilized the Biolog EcoPlate^TM^ to investigate the relationship between bacterial carbon metabolism and environmental selectivity, demonstrating its effectiveness in assessing microbial communities in various environments, including plankton [19,20], sediment [21], sediment–water interfaces [22], groundwater [23], and soil [24].

Garland and Mills [25] initially introduced the concept of using community-level sole-carbon-source utilization patterns for the functional analysis of natural bacterial communities with GN plates developed by BIOLOG, Inc. Insam [26] later proposed a 96-well microplate (EcoPlate™) consisting of 31 environmentally relevant substrates plus a control, each replicated three times. Some authors who used the Biolog EcoPlate^TM^ noted several limitations including the bias in the method toward rapidly growing bacteria, the need to minimize the time between the sampling and inoculation of the microplates, and challenges in data analysis and interpretation [27,28]. However, using approaches like kinetic and multivariate analyses of the well color development data can help overcome these drawbacks and enhance understanding of bacterial responses [27,29,30]. Overall, the method proves particularly powerful for community comparisons [27].

Our hypothesis was that, in line with global-warming trends, the mean water temperature of lakes would increase in correlation with air temperatures. We investigated how the bacterioplankton metabolism responds to temperature and other seasonal environmental factors, particularly nutrient loads, within the lakes. Finally, we discuss the implications of our findings in the context of climate change, speculating on the effects of global warming on temperature shifts in high-mountain lakes. To examine the link between regional warming and bacterial metabolism, we employed the Biolog EcoPlate™ assay combined with various functional metrics. This approach allowed us to evaluate its effectiveness in rapidly detecting the biological responses of high-mountain lakes to changes in water and air temperatures.

## 2. Materials and Methods

### 2.1. Study Sites and Sampling

**Figure 1:**
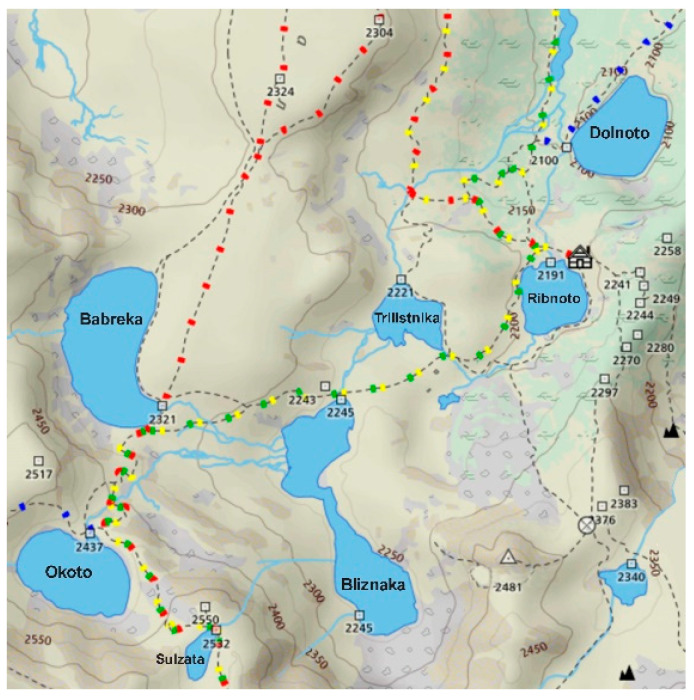
Map of the Seven Rila Lakes cirque, northwest Rila Mountains, Bulgaria with the highest situated Sulzata (42°11′50.0″ N 23°18′38.9″ E), Okoto (42°11′57.5″ N 23°18′23.1″ E), and Bubreka (42°12′19.6″ N 23°18′26.4″ E) Lakes.

Three glacial lakes in the northwestern region of Rila Mountain (Bulgaria), Sulzata (Sul), Okoto (Oko), and Bubreka (Bub), were selected as sampling sites (Figure 1).

Sul Lake is the smallest of the three, situated at 2535 m above sea level (a.s.l.) with an area of 0.7 ha and max depth of 4.5 m. The Oko and Bub Lakes are located at 2440 m a.s.l. and 2282 m a.s.l., respectively, with areas and max depths of 6.8 ha and 8.5 ha, and 37.7 m and 28.0 m. The lakes are not interconnected, each draining into the lower-lying Bliznaka Lake, which was not included in this study.

Water samples from 3 points of the lake littoral were collected during the years 2015 (June and August), 2022 (October), 2023 (August and October), and 2024 (June, August and October). Water samples for microbiological analysis were collected in sterile equipment, and were stored and transported to the laboratory at 4 °C.

### 2.2. Environmental Parameters

Dissolved oxygen (DO; mg/L), water temperature (T; °C), electrical conductivity (EC, μS/cm), and pH were measured in situ using WTW (Germany) and Hanna (Romania) handheld meters, following Bulgarian State Standards: [31,32,33]. Water samples were filtered through glass fiber filters (Whatman GF/F; 0.7 μm, Buckinghamshire, United Kingdom) before analyzing dissolved organic carbon (DOC in mg/L; TOC-5000, Shimadzu, Japan) and its spectral properties at wavelengths of 250 nm, 254 nm, 365 nm, and 436 nm. Additionally, ammonium nitrogen (NH_4_-N in µg/L) [34], nitrate nitrogen (NO_3_-N in µg/L) [35], and phosphate phosphorus (PO_4_-P in µg/L) [36] were analyzed from filtered samples. Unfiltered samples were used for measuring total nitrogen (TN in µg/L) [35] and total phosphorus (TP in µg/L) [36]. Chlorophyll-a (Chl-a; μg/L) concentration was determined according to [37]. All colorimetric analyses were performed using a CECIL CE 3021 spectrophotometer (Cambridge, UK).

The examined DOC spectral properties included

- SUVA_254_—indicative of DOC aromaticity [38], calculated according to Equation (1):
  (1) SUVA254=A254DOC
  where A is the absorbance at 254 nm measured in inverse meters (m^−1^) divided by the DOC concentration measured in milligrams per liter (mg/L);
- E_2_/E_3_: The ratio of absorbencies at 250 nm to 365 nm indicating the molecular weight of DOC. The increase of E_2_/E_3_ values correlates with the decrease of DOC aromaticity and molecular weight [39];
- E_2_/E_4_: The ratio of absorbencies at 254 nm to 436 nm provides insights into the predominant source of DOC in lakes [40]. E_2_/E_4_ values within the range of 4.0 to 11.0 suggest that DOC originates from terrestrial and/or macrophyte sources [40];
- DOC/TN: The ratio was determined according to [41], showing the DOC origin; DOC/TN ≤10: algal origin; DOC/TN >20: terrestrial origin, and 12 < DOC/TN < 17: multiple DOC sources.

### 2.3. Bacterial Metabolic Profiles

Biolog EcoPlates^TM^ (Biolog Inc., Hayward CA, USA) were used to assess the metabolic capacity of heterotrophic bacterial communities [42]. Each microplate contains 31 carbon sources (CSs) categorized into five carbon guilds (Table 1): carbohydrates, polymers, carboxylic acids, amino acids, and amines [43].

The microplates were inoculated with 120 μL of lake water and were incubated at 22 ± 1 °C in the dark. The optical densities (ODs) of the wells were measured every 12 h for five days at 590 nm using Microplate Reader LKB 5060-006 with the DV990 “Win 6” software package. Kinetic data were used to calculate the area under the curve (AUC; square units: SU; Equation (2)), which facilitated the evaluation of the following endpoints: (1) the average well color development (AWCD) as a measure of mean bacterial metabolic activity (Equation (3)); (2) the community level physiological profile (CLPP) illustrating the spectrum and rate of utilizable CSs; and (3) the functional diversity (Shannon–Weaver diversity index; H′; Equation (4)) and evenness (Pilou’s index; E; Equation (5)). The applied equations were

(2) AUC=∑ODn+ODn+12×tn+1−tn

where OD_n_ and OD_n+1_ represent the optical densities at two consecutive measurements at times t_n_ and t_n+1_ [17].

- AWCD was calculated as
  (3) AWCD=∑AUCiN
  where AUC_i_ is the area under the curve of the i-th CS, and N is the number of EcoPlate^TM^ CSs (N = 31) [25];
- The Shannon–Weaver (H′) and Pilou’s (E) indices were calculated as
  (4) $$H^{\prime }=\sum p_{i}\times \ln\left(p_{i}\right)$$
  (5) $$E=\frac{H^{\prime }}{logN}$$
  where p_i_ is the ratio between the AUC of the i-th CS to the sum of the AUC of all CSs [44].

Before the calculation of AWCD, CLPP, H′ and E, the control AUC was subtracted from the AUC of each CS.

### 2.4. Statistical Analyses

Each data point in the paper represents the mean value of the respective parameter ± standard deviation. A one-way ANOVA, followed by Tukey’s test, was performed to examine the significance of differences in the means of the studied parameters. Cluster analysis (method: group average—unweighted pair-group; similarity index: Euclidean distance; Box–Cox transformed data) was performed to evaluate the similarity between lake environments. Pearson correlation analysis was used to assess the relationships between the studied metrics. A redundancy analysis on Box–Cox transformed data, followed by a Monte Carlo test, was employed to ordinate the CLPPs and identify significant CLPP–environment relationships. The Similarities Percentages Procedure (SIMPER) of ordinated CLPP groups was used to determine the overall intragroup/intergroup Bray–Curtis similarity/dissimilarity. All statistics were performed with the NCSS (Kaysville, UT, USA; cluster analysis) [45] and PAST version 4.03 [46] software packages at a significance level of *p* < 0.05.

## 3. Results

### 3.1. Lake Environments

During the study period, several physicochemical parameters of water were measured (Table S1). The water temperature ranged from 2.6 °C (Sul; June 2015) to 20.9 °C (Bub; August 2024). The lakes were pH-neutral, with a mean pH of 7.32. Based on Chl-a content, the lakes were classified as oligotrophic (Oko and Bub) and mesotrophic (Sul). The primary production was phosphorus-limited (TN:TP ratio = 35.33, on average). DOC concentrations varied between a minimum of 1.57 mg/L and a maximum of 8.25 mg/L. DOC spectral properties were measured in 2023 (August) and 2024 (June, August and October), revealing that DOC consisted of hydrophilic, low-molecular-weight compounds with low aromaticity (SUVA_254_ = 0.01; E_2_/E_3_ = 2.96) and primarily originated from phytoplankton (E_2_/E_4_ = 1.74). The ratio DOC/TN (2.48 ± 1.47, on average) further confirmed the algal origin of DOC.

**Figure 2:**
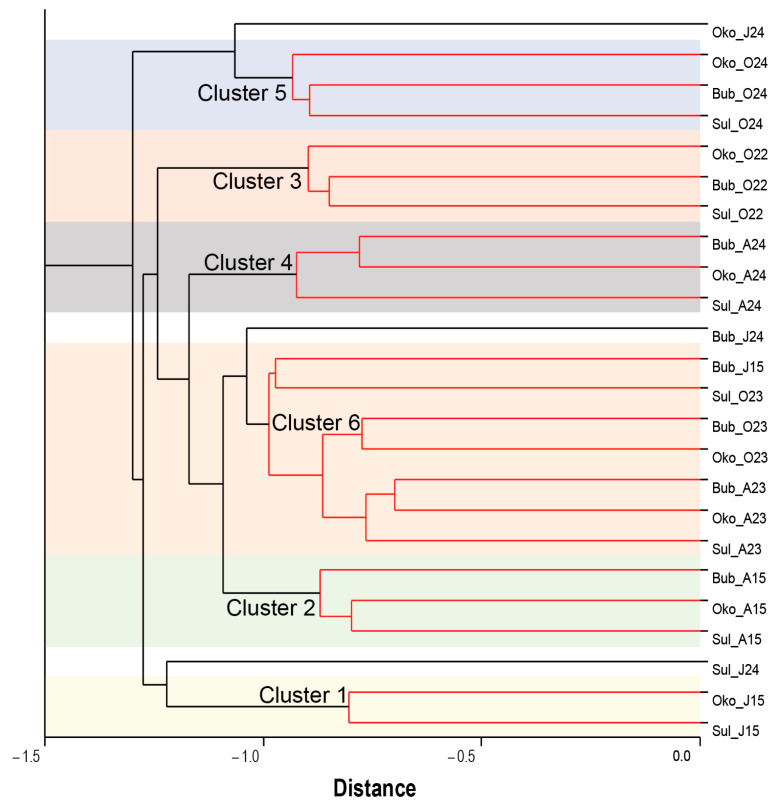
Dendrogram of the clustering analysis (group average—unweighted pair-group) illustrating the grouping of Sulzata (Sul), Okoto (Oko), and Bubreka (Bub) Lake environments (clusters) based on similarity in water characteristics (Euclidean distance) during June (J), August (A), and October (O) in the years 2015 (15), 2022 (22), 2023 (23), and 2024 (24).

In this study, cluster analysis (CA) was employed to assess similarities in the physical environments of the lakes (Figure 2; Table S1).

CA revealed that the lake environments were grouped into six clusters, each containing environments with similar features. However, CA was less effective in clustering lake environments from June 2024. To analyze the water-quality characteristics of each cluster, box plots of water parameters were generated and are presented in Figure S1. Cluster 1 exhibited the lowest median values for temperature (2.95 ± 0.38 °C), pH (6.66 ± 0.19), Chl-a (0.53 ± 0.08 µg/L), EC (6.98 ± 0.45 µS/cm), and NH_4_-N (24.90 ± 2.24 µg/L). The lowest nutrient concentrations were observed in different clusters: nitrogen (NO_3_-N: 93.54 ± 17.09 µg/L) in Cluster 2, TP (20.40 ± 0.51 µg/L) and DOC (0.57 ± 0.46 mg/L) in Cluster 4, and PO_4_-P (10.00 ± 0.17 µg/L) in Cluster 5. In contrast, the highest values for pH (7.78 ± 0.07), T (17.80 ± 0.55 °C), and DO (11.21 ± 0.37 mg/L) were associated with Clusters 3, 4, and 5, respectively. Maximum nitrogen concentrations were observed in Clusters 5 (NO_3_-N: 820.00 ± 42.22 µg/L; NH_4_-N: 112.20 ± 3.40 µg/L) and Cluster 1 (TN: 1554.25 ± 32.47 µg/L). The highest phosphorus (PO_4_-P: 39.20 ± 1.68 µg/L; TP: 60.00 ± 2.91 µg/L), DOC (8.25 ± 0.39 mg/L) and Chl-a (3.30 ± 0.57 µg/L) concentrations were found for Cluster 3.

**Figure 3:**
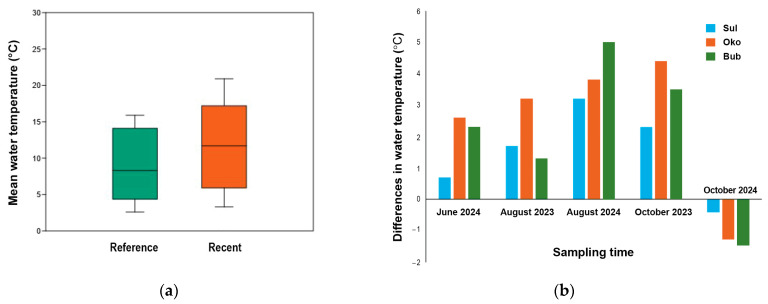
Lake water temperature presented as (**a**) mean water temperature (°C) for the reference (2015 and 2022; n = 27) and recent (2023 and 2024; n = 45) years, and (**b**) temperature differences in June (n = 18), August (n = 27) and October (n = 18) for Sulzata (Sul), Okoto (Oko), and Bubreka (Bub) Lakes.

Water temperature was used as an indicator of the effects of air warming on lake environments. Its values from 2023 and 2024 were compared to those recorded in 2015 (June and August) and 2022 (October) (Figure 3). The mean water temperature during the reference period was 8.8 ± 4.9 °C, while in recent years it has risen to 12.9 ± 5.6 °C or 11.4 ± 5.8 °C when accounting for the colder October 2024 (Figure 3a). In particular, the lake temperature increased by 1.9 °C in June 2024, 3.0 °C in August 2023, and by 3.4 °C in October 2023. However, it decreased by 1.05 °C in October 2024. The measured increase in water temperature showed a linear correlation with rising air temperatures recorded from the nearest meteorological station (Musala peak in Rila Mountains) during the same observation period (Pearson correlation: r = 0.67; *p* ≤ 0.001; Table S2) (Figure S2). The negative temperature difference observed in October 2024 (Figure 3) may be attributed to either the chosen reference year or the later sampling date.

Rainfall decreased over time (Figure S3) and demonstrated a weak, insignificant correlation with water temperature (Table S2). Pearson correlation analysis also indicated strong relationships between water temperature, and DO, DOC, and Chl-a (negative correlations), as well as a positive correlation with SUVA_254_ (Table S2).

### 3.2. Bacterial Metabolic Activity and Functional Profiles

#### 3.2.1. AWCD

**Figure 4:**
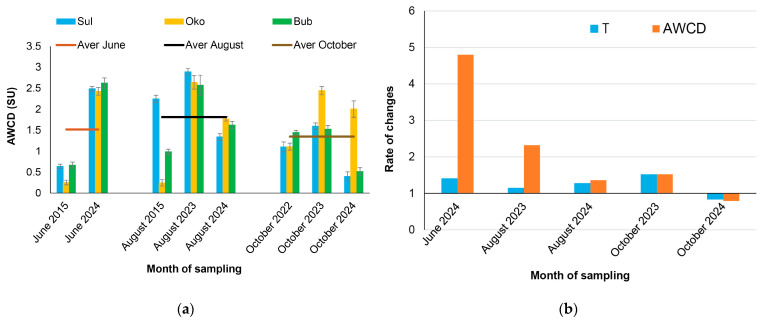
Bacterial metabolic activity shown as: (**a**) monthly average well color development (AWCD) in Sulzata (Sul), Okoto (Oko), and Bubreka (Bub) lakes (n = 3 per lake); and (**b**) rates of change in AWCD and water temperature (T) in recent years compared to reference years, presented as means across all lakes. In panel (**a**), the lines represent the average AWCD values for all lakes for each corresponding month.

The lake metabolic activity (AWCD) varied by sampling time and lake (Figure 4), with a minimum value recorded in June 2015 (0.519 ± 0.23 SU) and a maximum value in August 2023 (2.70 ± 0.17 SU).

A general trend of increasing AWCD over time was observed across the lakes, although some lake-specific variations were noted. The rate of increase was particularly pronounced in June 2024, while a decline in AWCD was only found in October 2024. Pearson correlation analysis revealed positive correlations between AWCD and T (r = 0.36; *p* = 0.01) and TN (r = 0.37; *p* = 0.01), while negative correlations were observed between AWCD and E_2_/E_4_ (r = −0.50; *p* = 0.02) and Chl-a (r = −0.34; *p* = 0.02) (Table S2). The average increase in water temperature over recent years (excluding October 2024) was between 1.1 and 1.5 times per sampling month, while changes in AWCD varied from 1.4 (August 2024) to 4.8 (June 2024) times, indicating significant effects of warming on bacterial metabolic activity, particularly in June 2024.

#### 3.2.2. CLPPs

**Figure 5:**
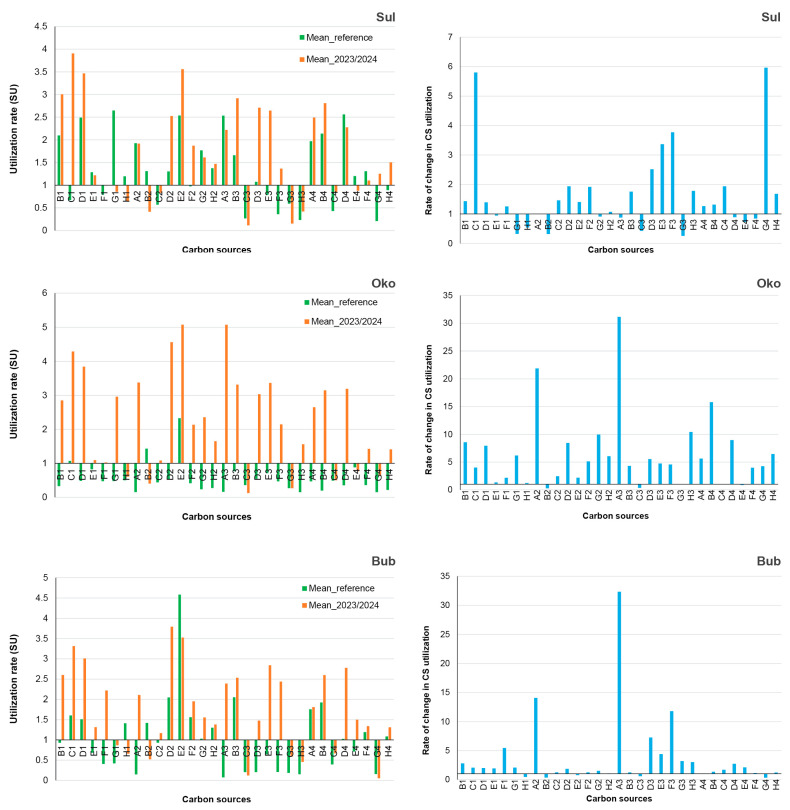
Community-level physiological profiles (CLPPs) of bacteria from Sulzata (Sul), Okoto (Oko), and Bubreka (Bub) Lakes, expressed in the left column as means of reference (Mean_reference) and recent years (Mean_2023/2024), and in the right column as differences between the two monitoring periods.

AWCD indicated the average capacity of bacteria to utilize 31 carbon sources (CSs), with the rates of CS utilization defining the functional profiles of bacterial communities (CLPPs). CLPPs varied over time and among lakes; however, certain CSs (C3, G3, H3, C4, and G4) were poorly utilized (<50% of AWCD), while others, such as C1, D1, and E2, were highly preferred (>150% of AWCD) by bacteria throughout the study period (Figure 5).

The transition from the reference period to recent years altered the utilization rates of certain CSs, with decreases noted for G1, H1, B2, and C2, while the utilization of D2 and E3 increased. Additional lake-specific changes in CLPPs were also observed. This intricate dynamic of the CLPPs was accompanied by relatively minor changes in bacterial functional diversity (H′; Table 2), primarily related to changes in the evenness of CS utilization rates (Pearson correlation; r = 0.99; *p* < 0.0001), rather than in the richness of utilizable CSs.

**Figure 6:**
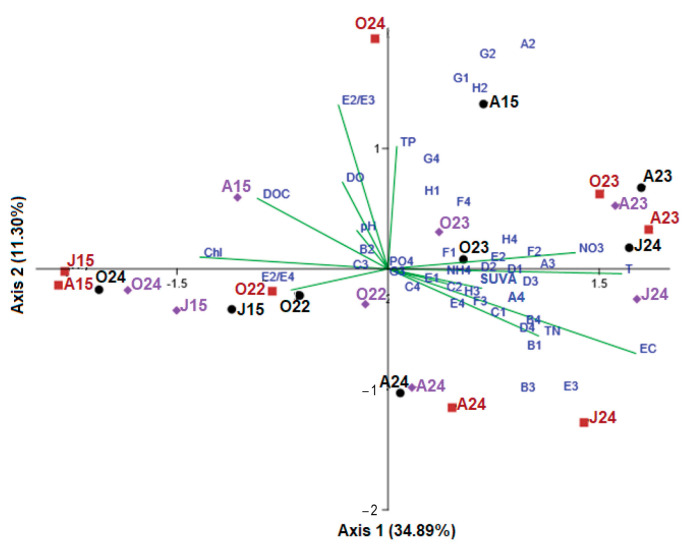
RDA plot of CSs’ (blue dots) distribution in relation to sampling months and lakes (black dot—Sul; brown squire—Oko and violet diamond—Bub) and correlations with environmental variables (lines).

To evaluate the influence of water parameters on the functional structure of bacterial metabolism, CLPPs were ordinated using redundancy analysis (RDA) (Figure 6). Monte Carlo tests for the first and all canonical axes were highly significant (*p* = 0.017), indicating that these environmental parameters critically influence bacterial-community functioning. The first two axes accounted for 34.9% and 11.3% of the variation in the CLPPs, respectively, while the first four significant axes explained over half of the CLPP variation (57.7%), increasing to 73% when all of the significant axes were included. For clearer representation of the CLPPs ordination, the first two axes were used for plotting.

RDA identified two main groups of CLPPs. Group A included the bacterial functional profiles from August and October 2023, June and August 2024, and August 2015 (Sul), exhibiting an intragroup similarity of 70.1%. Group B comprised CLPPs from June and August 2015 (Oko and Bub), October 2022 and 2024, with an intragroup similarity of 44.5%. The dissimilarity between the two groups was 61.4%. Group A showed a higher Shannon diversity index (H′ = 3.08 ± 0.20) compared to group B (2.91 ± 0.24), and the difference between the two groups was significant (F = 3.93, *p* = 0.007). The increased bacterial functional diversity in group A was associated with higher evenness in CS utilization rates (E = 0.70 ± 0.11) compared to group B (E = 0.61 ± 0.13).

The utilization rates of 24 out of the 31 tested CSs were linked to the specific bacterial functional profiles of group A. These included carbohydrates (G1, H1, A2, D2, E2, G2, H2) with varying utilization rates: some decreased (G1, H1, E2 and H2), while others increased (A2 and D2) compared to the reference period. Additionally, non-proteinogenic (B1, F2, A3, B3, D3, E3, F3) and proteinogenic (B4 and D4) carboxylic acids became more favorable CSs in recent years, alongside polymer C1.

RDA incorporated 14 environmental factors (T, pH, DO, EC, PO_4_-P, TP, NH_4_-N, NO_3_-N, TN, Chl-a, DOC, SUVA_254_, E_2_/E_3_, and E_2_/E_4_), and it was found that the key factors positively influencing the bacterial functional profiles in Group A included water temperature (T), electrical conductivity (EC), and the concentrations of total phosphorus (TP) and nitrogen (NO_3_-N and TN). In contrast, dissolved organic carbon (DOC), its spectral properties (E_2_/E_3_ and E_2_/E_4_), and chlorophyll-a (Chl-a) had negative effects. In Group B, bacterial functional profiles were primarily influenced by positive effects from DOC and Chl-a concentrations, and negative effects by water temperature and concentrations of nitrogen and phosphorous.

## 4. Discussion

### 4.1. Temporal Changes in Lake Environments

High-mountain lakes are unique environments, characterized by harsh air and water temperatures, prolonged ice cover, a short ice-free season, and relatively small catchments with sparse vegetation. Precipitation serves as the primary source of a water supply. In this context, climate change significantly impacts the hydrochemistry and biology of these lakes. Meteorological records from 2015 to 2024 [47] indicated a trend of rising air temperatures (Figure S2), alongside a decline in precipitation, particularly in recent years (Figure S3). These climatic shifts have affected water temperature, with increases observed in 2023 and 2024 (except for October), averaging 2.6 °C compared to the reference period (Figure 3). Pearson correlation analysis revealed a strong positive correlation between air temperature and lake water temperature, while the correlation with rainfall was low and insignificant (Table S2). Similar relationships between the air temperature, precipitation, and water temperature of Sul, Oko, and Bub were also reported by Nikolova et al. [48].

The impact of climate on lake life is mediated through changes in water temperature, which regulate many chemical and biological processes and strongly influence lake characteristics [49]. Water temperature showed a significant correlation with DOC (negative) and SUVA_254_ (positive), likely affecting the solubility of organic carbon and its biochemical composition. The decline in DOC with rising water temperature was primarily linked to enhanced biological activity [50,51] and photooxidation [52,53] during the longer ice-free season.

Although the studied lakes differ morphometrically and hydrologically, we expected that they would respond to climate forcing in a broadly similar manner. In this context, cluster analysis (Figure 2) revealed month groupings of lake environments, indicating that seasonal variations outweighed interannual differences. However, lakes from different years formed distinct clusters with varying distance between them. Notably, the physical environments of the lakes from June 2015 (Cluster 1) and June 2024 (undefined cluster) exhibited the greatest divergence (except Sul), suggesting that interannual differences in June were more pronounced than in other sampling months. We speculate that these differences result from earlier snowmelt in recent years [54] and its consequent effects. For instance, Ivanov et al. [54], determined that the melting period of Sul Lake occurred between 25 July and 15 August. Boteva et al. [55] also reported thick ice cover on Sul Lake on 4 July 2006. However, more recent observations (24 June 2015 and 14 June 2024; personal data) found that the ice cover had completely melted by those dates.

### 4.2. Bacterial Response to Environmental Changes over Time

Warming of water can alter chemical conditions and nutrient balance in lakes, complicating predictions about effects on aquatic life. To better forecast the impact of global warming on lake ecosystems, it is essential to investigate key factors influencing primary productivity and food-web stability. One such factor is the microbial loop, which plays a significant role in processes such as organic matter degradation, nutrient cycling, and energy transfer to higher trophic levels [56]. This study examined bacterial metabolic activity and CS utilization rates in the context of the gradual warming of lake water over recent years.

#### 4.2.1. Bacterial Metabolic Activity (AWCD)

It is widely recognized that bacterial metabolic rates increase with higher temperatures within the mesophilic range [57,58]. However, the effects of warming on natural psychrotolerant bacterial communities, which dominate polar regions and alpine ecosystems [59], have been less extensively studied.

In this study, bacterial metabolic activity increased and was positively related to water temperature, with maximum effects observed in June 2024 (Figure 4). In this context, the greatest effects of global warming on high-mountain lake life are expected to occur at the onset of the growing season, when water chemistry is strongly influenced by snowmelt. During this period, there is an increase in water and nutrients influx, enhanced penetration of solar radiation, the initiation of the phytoplankton growing season, and a growing complexity of trophic interactions [60,61]. This assumption requires further investigation to confirm, enabling us to rule out any potential exceptions to the trends observed in other sampled months.

Our results are consistent with previous findings indicating increased bacterioplankton activity in early spring, coinciding with a substantial input of labile allochthonous dissolved organic matter from lake catchments [62]. Additionally, laboratory experiments by Christoffersen et al. [63] confirmed that climate-induced changes in nutrient loading play a primary role in shaping lake bacterial communities, with temperature effects being most pronounced at low nutrient levels, rather than as a direct result of climate warming. In our study, nutrient effects on AWCD (across lakes and the overall time of study) were evident through significant positive relationships between AWCD and TN, as well as negative relationships between AWCD and Chl-a and DOC spectral properties (algal origin; E_2_/E_4_, and DOC/TN). These relationships suggest bacterial pressure on both primary and extracellular primary production. Conversely, the negative relationship between DOC and water temperature (r = −0.62; *p* = 0.0001) indicated its decline in DOC with rising temperatures, likely due to biological or physicochemical oxidation.

#### 4.2.2. Bacterial Community Functional Profiles (CLPPs)

Bacteria can adjust their metabolic niche to effectively adapt to the available nutrients in the environment [64]. One hypothesis posits that bacteria would shift their metabolism toward utilizing CSs to which they were best adapted at any given moment during the study, and that the similarity or dissimilarity between CLPPs could reflect an intrinsic bacterial response to environmental changes. Bacterial functional profiles varied across seasons and years, with interannual changes being of greater interest for the focus of the study. The RDA conducted on CLPPs (Figure 6) separated the bacterial functional profiles of 2023 and 2024 (group A), positioning them on the right side of the ordination plot, distinctly away from the profiles of the reference years (group B). This pattern of interannual segregation indicated a strong functional dissimilarity between the two groups, with group A exhibiting higher functional diversity (H’) and significantly differing from group B.

The shifts in CLPPs over time occurred in two main directions: (1) changes in the utilization rates of CSs and (2) changes in the preferred CSs based on their chemical moiety (Figure 5). CLPP ordination identified 24 out of 31 CSs, whose utilization rates reflected shifts in bacterial functional profiles from the reference period to more recent times. Most of these CSs were carbohydrates (G1, H1, A2, D2, E2, G2, and H2; 7 out of 9) as well as non-proteinogenic (B1, F2, A3, B3, D3, E3 F3, and H3; 8 out of 10) and proteinogenic (A4, B4, D4, E4, and F4; 5 out of 6) carboxylic acids. A consistent shift in CS utilization was observed, transitioning from a preference for carbohydrates during the reference period to a preference for carboxylic acids in recent years. Additionally, during the reference period, amino acids were prominently utilized in Sul Lake, while polymers were favored in the Oko and Bub Lakes. These utilization patterns remained stable over time. Numerous studies have shown that carbohydrates and amino acids constitute a significant portion of lake DOC, as they are key components of algal and plant exudates [65,66,67,68,69,70,71] or originate from the degradation of dead organic matter [52]. Additionally, several studies have reported seasonal variation in bacterial-substrate preferences, indicating that carbohydrates are predominantly utilized during the clear-water phase, while amino-acid consumption increases during periods of algal proliferation [65,70,71,72]. Grover and Chrzanowski [73] also found greater amino-acid utilization during cooler months compared to warmer ones in their study of four lakes in Canada and the USA. In this context, the preferential utilization of amino acids in Sul Lake can be linked to lower water temperatures and the higher phytoplankton biomass (Chl-a) observed during both the reference period (2.44 ± 3.31 mg/L; particularly in October 2022) and the recent (4.37 ± 2.98 mg/L) sampling periods. Overall, this study demonstrated that the transition from the reference period to recent years resulted in a shift in bacterioplankton metabolism, with a notable preference for the utilization of carboxylic acids over carbohydrates. We proposed that this metabolic shift was linked to changes in the composition of labile organic carbon. Previous studies have shown that both terrestrial and aquatic plants exude carboxylic acids in response to environmental or anthropogenic stress [74,75,76,77]. In human-impacted environments, researchers have also observed shifts in bacterial metabolism from carbohydrates to carboxylic acids [24,78,79], supporting the hypothesis that bacterial activity and functional diversity are shaped by the availability and quality of ecosystem resources [80]. In light of these findings, we suggest that primary producers in the lakes have been subjected to thermal or other environmental stressors (e.g., increased solar radiation during the longer ice-free season), resulting in an altered exudate composition. Lake bacterioplankton appear to have adapted to these shifts by modifying their metabolism to utilize the most readily available carbon sources. The RDA confirmed that water temperature in recent years is a major factor influencing bacterial metabolism, alongside nutrient availability and nitrogen- and phosphorus-containing nutrients (indicated by EC, NO_3_-N, TN, and TP). In contrast, the influence of DOC and phytoplankton biomass (Chl-a) has diminished. Notably, approximately 46% of the variability in CLPP could be explained by the environmental factors examined in the RDA, while the remaining 54% likely depends on unmeasured variables. This underscores the need for future studies to broaden the range of environmental variables considered and to test the hypothesis of bacterial adaptivity to the available carbon pool.

## 5. Conclusions

Lake water temperature has increased over time, likely due to rising regional air temperatures, which have subsequently influenced the rate and range of bacterial metabolism. On average, bacterial metabolic activity has increased by 2–3 times, with a notable shift from a preference for carbohydrates to the utilization of carboxylic acids. We suggest that these changes in bacterial metabolism may amplify the effects of climate change, potentially undermining ecosystem resilience. Further investigation will address this hypothesis and the questions raised in the discussion.

## Figures and Tables

**Table 1:** Distribution of EcoPlate CSs in carbon guilds according to Weber and Legge [43].

Carbohydrates	Carboxylic Acids	Amino Acids	Polymers
G1: D-Cellobiose	B1: Pyruvic acid methyl ester	A4: L-Arginine	C1: Tween 40
H1: α-D-Lactose	F2: D-Glucosaminic acid	B4: L-Asparagine	D1: Tween 80
A2: β-Methyl-D-Glucoside	A3: D-Galactonic acid γ-lactone	C4: L-Phenylalanine	E1: α-Cyclodextrin
B2: D-Xylose	B3: D-Galacturonic acid	D4: L-Serine	F1: Glycogen
C2: i-Erythritol	C3: 2-Hydroxy benzoic acid	E4: L-Threonine	
D2: D-Mannitol	D3: 4-Hydroxy benzoic acid	F4: β-Hydroxy-Glycyl-L-Glutamic acid	
E2: N-Acetyl-D-Glucosamine	E3: γ-Amino butyric acid		
G2: Glucose-1-Phosphate	F3: Itaconic acid	**Amines/amides**	
H2: D, L-α-Glycerol phosphate	G3: α-Keto butyric acid	G4: Phenylethylamine	
	H3: D-Malic acid	H4: Putrescine	

**Table 2:** Bacterial functional diversity (H′) and functional evenness (E) of community-level physiological profiles, expressed as mean and (standard deviation).

Lake	Index	June 2015	August 2015	October 2022	August 2023	October 2023	June 2024	August 2024	October 2024
Sul	H′	2.98 (0.12)	3.02 (0.14)	3.09 (0.15)	3.16 (0.09)	3.09 (0.16)	3.23 (0.09)	2.70 (0.23)	3.14 (0.06)
	E	0.63 (0.02)	0.66 (0.04)	0.71 (0.02)	0.76 (0.03)	0.71 (0.03)	0.81 (0.03)	0.48 (0.03)	0.74 (0.10)
Oko	H′	3.12 (0.00)	3.17 (0.00)	3.00 (0.14)	3.24 (0.09)	3.15 (0.12)	3.05 (0.01)	2.86 (0.16)	2.76 (0.16)
	E	0.73 (0.00)	0.76 (0.00)	0.65 (0.02)	0.83 (0.03)	0.75 (0.03)	0.68 (0.04)	0.56 (0.03)	0.51 (0.04)
Bub	H′	2.55 (0.17)	2.76 (0.16)	3.02 (0.17)	3.29 (0.10)	3.08 (0.18)	3.16 (0.10)	2.87 (0.19)	2.58 (0.08)
	E	0.41 (0.02)	0.51 (0.02)	0.66 (0.02)	0.87 (0.03)	0.70 (0.03)	0.76 (0.04)	0.57 (0.03)	0.42 (0.03)

## Data Availability

The original contributions presented in this study are included in the article/supplementary material. Further inquiries can be directed to the corresponding author.

## Supplementary Materials

The following supporting information can be downloaded at: https://www.mdpi.com/article/10.3390/microorganisms13061375/s1: Table S1. Physicochemical characteristics of Sulzata (Sul), Okoto (Oko), and Bubreka (Bub) Lake water, measured in June and August 2015 (J15, A15), October 2022 (O22), and June, August, and October 2024 (J24, A24, O24) and (standard deviation); Figure S1. Box plots illustrating the distribution of water parameter values across clusters, as defined in Figure 3; Figure S2. Trends in the average air temperature for June, August, and October from 2015 to 2024, expressed as a 3-point average; Figure S3. Trends in the average rainfalls for June, August, and October from 2015 to 2024, expressed as a 3-point average; Table S2. Pearson correlation analysis of water and climatic parameters.

## Abbreviations

The following abbreviations are used in this manuscript:

AUC	Area under the curve
AWCD	Average well color development
Bub	Lake Bubreka
CA	Cluster analysis
Chl-a	Chlorophyll-a
CLPP	Community level physiological profile
CSs	Carbon sources
DO	Dissolved oxygen
DOC	Dissolved organic carbon
EC	Electrical conductivity
NH_4_-N	Ammonium nitrogen
NO_3_-N	Nitrate nitrogen
Oko	Lake Okoto
PO_4_-P	Phosphate phosphorus
RDA	Redundancy analysis
Sul	Lake Sulzata
SUVA_254_	Specific ultraviolet absorbance at 254 nm
T	Temperature
TN	Total nitrogen
TP	Total phosphorus
