# Supplementary material for: Bacterial Metabolic Activity of High-Mountain Lakes in a Context of Increasing Regional Temperature
Source: Microorganisms. 2025 Jun 13;13(6):1375. doi: 10.3390/microorganisms13061375 (PMC12196075; doi:10.3390/microorganisms13061375)
Supplement: Supplementary file 1 [file microorganisms-13-01375-s001.zip › microorganisms-3645540-supplementary.pdf]

Table S1. Physicochemical characteristics of Sulzata (Sul), Okoto (Oko), and Bubreka (Bub) lake water, measured in June and August 2015 (J15, A15), October 2022 (O22), and June, August, and October 2024 (J24, A24, O24) and (standard deviation).

Sample	T (°C)	pH	DO (mg/L)	PO_4-P (µg/L)	NH_4-N (µg/L)	NO_3-N (µg/L)	TP (µg/L)	TN (µg/L)	EC (µS/cm)	Chl-a (µg/L)	DOC (mg/L)	$SUVA_{254}$	E_2/E_3	E_2/E_4	TN:TP	DOC/TN
**Sul_J15**	2.6 (0.10)	6.32 (0.04)	9.3 (0.06)	20 (2.9)	30 (4.5)	400 (23.2)	50 (6.0)	1500 (97)	6 (1.0)	0.36 (0.02)	ND	ND	ND	ND	30	ND
**Sul_A15**	14.6 (0.15)	7.6 (0.07)	7.6 (0.06)	20 (2.5)	30 (0.6)	160 (6.8)	40 (5.0)	300 (30)	9 (2.0)	0.71 (0.08)	ND	ND	ND	ND	7.5	ND
**Sul_O22**	5.4 (0.06)	8.1 (0.15)	9.6 (0.10)	40 (4.0)	ND	200 (16.6)	70 (7.5)	300 (84)	22 (2.2)	6.26 (0.06)	9.7 (0.75)	ND	ND	ND	4.28	32.33
**Sul_A23**	16.3 (0.10)	7.02 (0.07)	9.8 (0.06)	20 (3.1)	30 (2.0)	500 (20.0)	50 (4.0)	1200 (95)	18 (0.0)	2.76 (0.03)	2.04 (0.70)	0.007	2.17	0	24	1.69
**Sul_O23**	7.7 (0.15)	6.95 (0.05)	8.4 (0.10)	30 (2.5)	50 (2.5)	500 (7.5)	80 (6.0)	1200 (83)	19 (1.0)	7.99 (0.04)	5.95 (0.40)	ND	ND	ND	15	4.96
**Sul_J24**	3.3 (0.15)	7.98 (0.10)	10.5 (0.10)	17 (2.0)	60 (4.7)	600 (16)	26 (6.0)	1800 (38)	13.3 (0.0)	0.63 (0.06)	6.67 (0.35)	0.001	1.143	0.8	69.23	3.7
**Sul_A24**	17.8 (0.10)	7.28 (0.10)	7.54 (0.05)	16 (1.0)	50 (1.5)	700 (20.5)	23 (8.0)	1300 (71)	31 (0.0)	2.49 (0.10)	3.18 (0.26)	0.007	1.77	2.87	56.52	2.45
**Sul_O24**	5 (0.21)	7.45 (0.06)	11.21 (0.06)	10 (2.8)	110 (2.1)	600 (14.8)	34 (5.0)	1100 (100)	21 (1.0)	6.09 (0.07)	1.1 (0.17)	0	0	0.5	32.35	1.01
**Oko_J15**	3.3 (0.21)	7 (0.06)	9.7 (0.06)	20 (2.6)	20 (1.1)	700 (17.8)	40 (3.0)	1600 (64)	8 (1.0)	0.71 (0.02)	ND	ND	ND	ND	40	ND
**Oko_A15**	13.6 (0.25)	7.5 (0.06)	8.1 (0.21)	20 (3.0)	20 (3.0)	50 (1.0)	30 (5.0)	400 (100)	13 (1.0)	0.36 (0.04)	ND	ND	ND	ND	13.33	ND
**Oko_O22**	6.3 (0.10)	7.7 (0.10)	11 (0.10)	40 (1.0)	20 (1.0)	30 (18.0)	60 (4.5)	500 (21)	28 (1.0)	2.48 (0.08)	7.05 (0.40)	ND	ND	ND	8.33	14.1
**Oko_A23**	16.8 (0.25)	7.05 (0.17)	9.7 (0.25)	20 (2.5)	30 (2.5)	700 (23.0)	ND	1460 (93)	22 (1.0)	0.59 (0.04)	1.47 (0.64)	ND	ND	ND	ND	1.01
**Oko_O23**	10.7 (0.25)	7.01 (0.11)	ND	20 (2.5)	30 (1.5)	800 (10.0)	60 (8.0)	1600 (80)	25 (1.0)	0.79 (0.06)	4.62 (0.32)	ND	ND	ND	26.67	2.88
**Oko_J24**	5.9 (0.15)	5.8 (0.06)	9.7 (0.15)	13 (1.5)	60 (2.5)	772 (9.6)	26 (5.5)	1483 (62)	46 (1.0)	0.79 (0.08)	6.81 (0.46)	0.0003	0.2	2	57.04	4.59
**Oko_A24**	17.4 (0.21)	7.36 (0.05)	7.13 (0.26)	16 (2.0)	50 (1.5)	772 (9.2)	20 (7.0)	1883 (78)	51 (1.0)	0.36 (0.05)	0.28 (0.36)	0.014	1.5	2	94.15	0.15
**Oko_O24**	5.03 (0.10)	7.02 (0.10)	11.41 (0.10)	10 (1.0)	110 (4.5)	870 (5.0)	33 (7.0)	1283 (112)	32 (1.0)	1.6 (0.05)	3.68 (0.35)	0.005	11	2.83	38.88	2.87

ND – not defined

Sample	T (°C)	pH	DO (mg/L)	PO_4-P (µg/L)	NH_4-N (µg/L)	NO_3-N (µg/L)	TP (µg/L)	TN (µg/L)	EC (µS/cm)	Chl-a (µg/L)	DOC (mg/L)	$SUVA_{254}$	E_2/E_3	E_2/E_4	TN:TP	DOC/TN
**Bub_J15**	9.4 (0.15)	7.1 (0.12)	9.8 (0.32)	20 (2.0)	50 (3.2)	50 (7.5)	50 (5.5)	1700 (130)	14 (2.0)	4.97 (0.07)	ND	ND	ND	ND	34	ND
**Bub_A15**	15.9 (0.15)	7.4 (0.12)	8.4 (0.15)	20 (1.5)	60 (1.5)	70 (6.5)	50 (6.5)	400 (77)	13 (1.0)	2.49 (0.02)	ND	ND	ND	ND	8	ND
**Bub_O22**	8.3 (0.15)	7.7 (0.15)	10.9 (0.15)	30 (1.5)	ND	300 (15.5)	50 (4.5)	200 (79)	28 (2.0)	3.3 (0.04)	8 (0.42)	ND	ND	ND	4	40
**Bub_A23**	17.2 (0.15)	7.28 (0.05)	9.6 (0.21)	20 (2.0)	40 (2.5)	ND	ND	ND	23 (1.0)	0.47 (0.06)	ND	ND	ND	ND	ND	ND
**Bub_O23**	11.8 (0.15)	7.05 (0.06)	8.7 (0.15)	20 (1.5)	40 (2.0)	700 (11.5)	50 (7.5)	1100 (62)	24 (0.0)	2.76 (0.08)	3.53 (0.26)	ND	ND	ND	22	3.21
**Bub_J24**	11.7 (0.10)	7.77 (0.10)	8.8 (0.06)	13 (1.0)	100 (2.5)	700 (11.5)	53 (6.5)	1850 (41)	25 (1.0)	0.95 (0.05)	6.5 (0.59)	0	0	0	34.9	1.76
**Bub_A24**	20.9 (0.15)	7.75 (0.06)	7.94 (0.05)	16 (1.5)	50 (2.0)	700 (13)	20 (4.5)	1100 (88)	26 (2.0)	0.53 (0.03)	0.57 (0.62)	0.059	1.064	2.12	55	0.29
**Bub_O24**	6.83 (0.21)	6.96 (0.06)	9.1 (0.10)	10 (2.0)	130 (3.0)	820 (8.5)	26 (7.5)	1000 (55)	23 (0.0)	3.28 (0.04)	2.13 (0.45)	0.008	2	3.6	38.46	1.52

ND – not defined

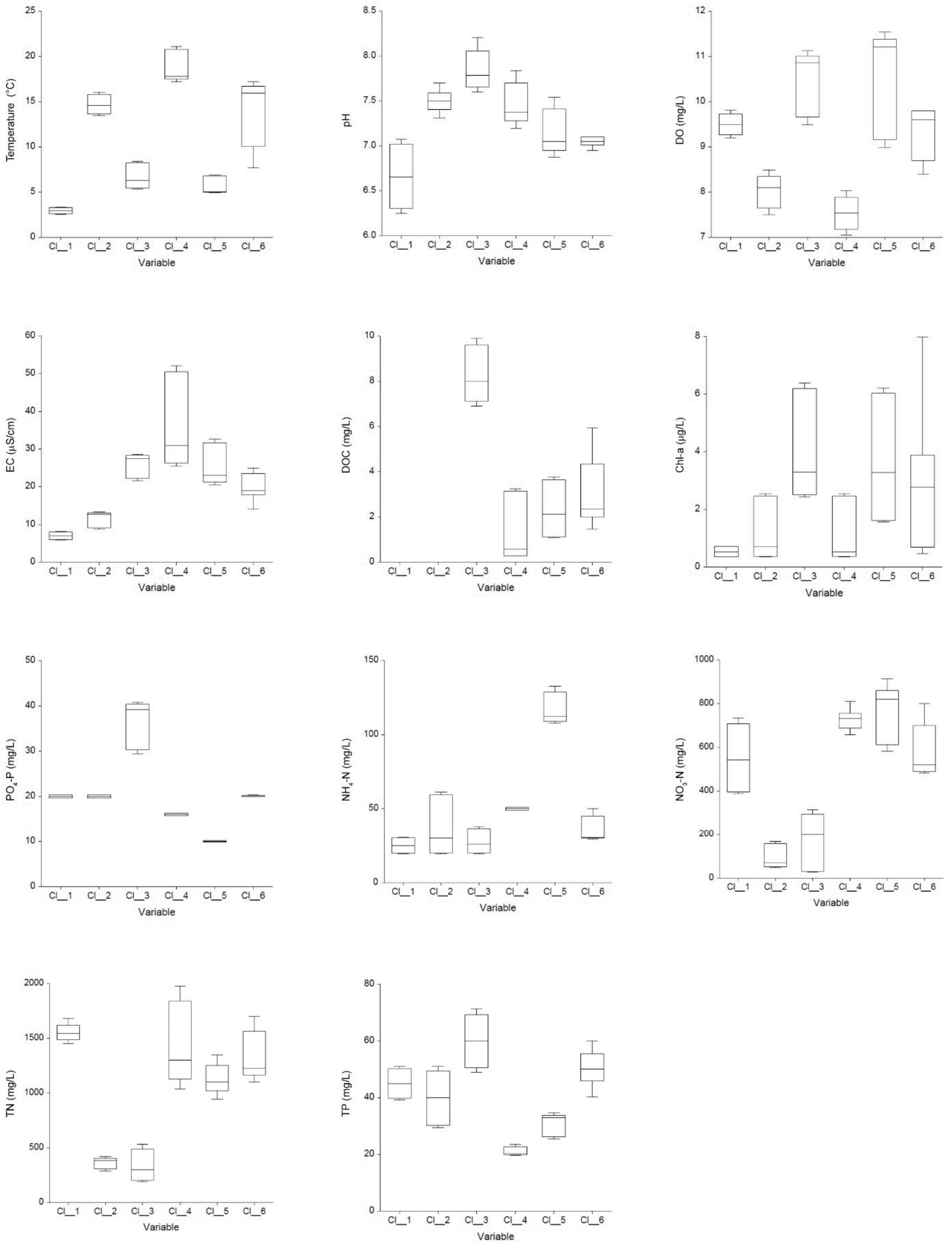

Figure S1. Box plots illustrating the distribution of water parameter values across clusters, as defined in Figure 3.

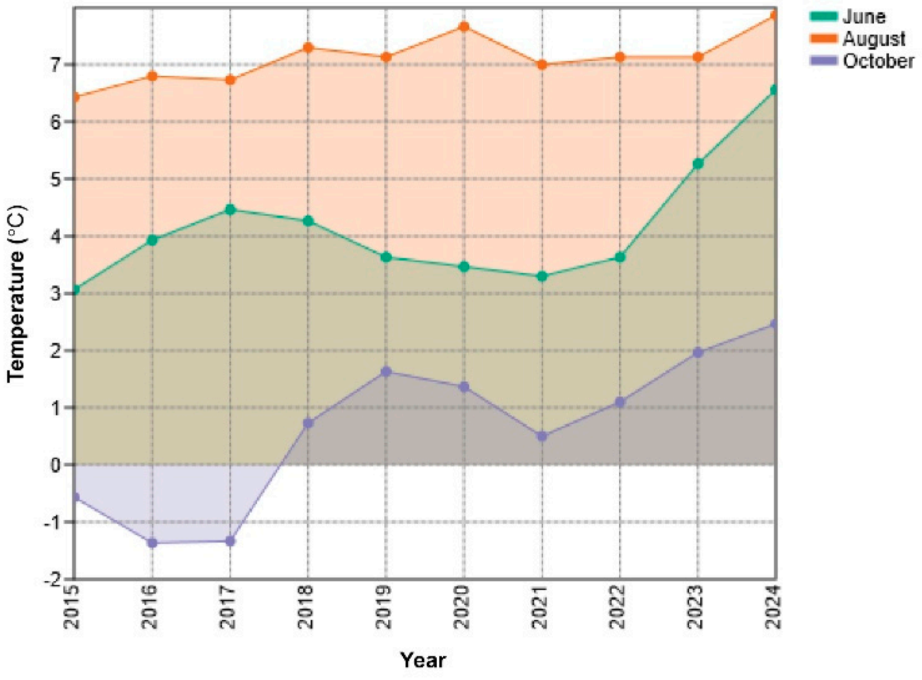

Figure S2. Trends in the average air temperature for June, August, and October from 2015 to 2024, expressed as a 3-point average.

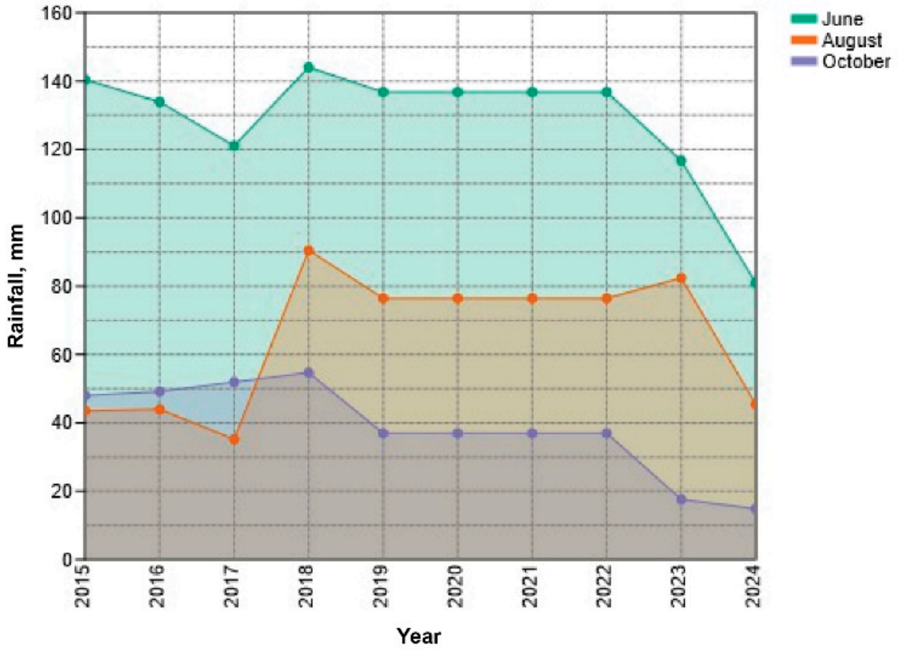

Figure S3. Trends in the average rainfalls for June, August, and October from 2015 to 2024, expressed as a 3-point average.

Table S2. Pearson correlation analysis of water and climatic parameters.

	water T	pH	DO	PO_4-P	NH_4-N	NO_3-N	TP	TN	EC	DOC	SUVA	E_2/E_3	E_2/E_4	Chl-a	DOC/ TN	Air T	Rainfall	AWCD
**water T**		0.18	0.00	0.34	0.09	0.06	0.08	0.73	0.17	0.00	0.00	0.37	0.92	0.04	0.08	0.00	0.17	0.01
**pH**	0.20		0.91	0.01	0.81	0.46	0.63	0.45	0.29	0.24	0.23	0.62	0.15	0.38	0.03	0.85	0.82	0.63
**DO**	-0.64	-0.02		0.41	0.09	0.02	0.23	0.42	0.71	0.05	0.04	0.08	0.26	0.16	0.05	0.00	0.00	0.73
**PO_4-P**	-0.14	0.35	0.12		0.00	0.00	0.00	0.00	0.33	0.00	0.28	0.21	0.10	0.01	0.00	0.00	0.09	0.36
**NH_4-N**	-0.26	0.04	0.27	-0.67		0.00	0.09	0.03	0.10	0.58	0.18	0.25	0.29	0.04	0.03	0.56	0.16	0.53
**NO_3-N**	0.30	-0.12	-0.38	-0.73	0.57		0.00	0.00	0.11	0.00	0.15	0.95	0.07	0.01	0.00	0.00	0.45	0.08
**TP**	-0.26	0.08	0.19	0.71	-0.27	-0.52		0.19	0.04	0.00	0.07	0.98	0.00	0.00	0.01	0.00	0.00	0.87
**TN**	0.05	-0.12	-0.13	-0.52	0.35	0.62	-0.20		0.21	0.14	0.78	0.20	0.10	0.06	0.00	0.01	0.07	0.01
**EC**	0.20	-0.16	-0.06	-0.14	0.25	0.26	-0.32	0.19		0.44	0.89	0.78	0.09	0.57	0.68	0.13	0.47	0.03
**DOC**	-0.62	0.21	0.35	0.62	-0.10	-0.51	0.59	-0.27	-0.14		0.02	0.83	0.34	0.13	0.00	0.03	0.94	0.91
**SUVA**	0.65	0.28	-0.47	0.26	-0.31	0.33	-0.41	0.07	0.03	-0.53		0.82	0.31	0.18	0.01	0.33	0.42	0.62
**E_2/E_3**	-0.21	-0.12	0.40	-0.29	0.27	0.02	0.01	-0.30	0.07	-0.05	-0.05		0.07	0.74	0.51	0.03	0.03	0.90
**E_2/E_4**	0.02	-0.33	-0.27	-0.38	0.25	0.41	-0.70	-0.38	0.39	-0.23	0.24	0.41		0.66	0.86	0.16	0.53	0.02
**Chl-a**	-0.29	0.13	0.21	0.37	0.31	-0.42	0.56	-0.29	-0.08	0.26	-0.31	-0.08	-0.10		0.06	0.00	0.00	0.02
**DOC/TN**	-0.32	0.39	0.36	0.72	-0.41	-0.63	0.44	-0.63	-0.08	0.70	-0.54	0.16	0.04	0.34		0.00	0.18	0.06
**Air T**	0.67	0.03	-0.50	-0.45	-0.09	0.47	-0.56	0.37	0.22	-0.38	0.23	-0.48	-0.33	-0.54	-0.55		0.00	0.00
**Rainfall**	0.20	0.03	-0.50	-0.24	-0.22	0.12	-0.45	0.27	-0.11	0.01	0.19	-0.48	-0.15	-0.49	-0.24	0.57		0.91
**AWCD**	0.36	-0.07	-0.05	-0.14	-0.10	0.28	0.02	0.37	0.32	0.02	-0.12	-0.03	-0.50	-0.34	-0.33	0.59	-0.02	
